# Domestic Violence Against Women and Their Mental Health Status in a Colony in Delhi

**DOI:** 10.4103/0970-0218.69266

**Published:** 2010-07

**Authors:** Alka S Vachher, AK Sharma

**Affiliations:** Department of Community Medicine, Vardhman Mahavir Medical College, India; 1 Lady Hardinge Medical College, New Delhi, India

**Keywords:** Domestic violence, mental health status, violence against women

## Abstract

**Background:**

Violence against women is a major public health and human rights issue in the world today. This study was conducted to assess the consequences of domestic violence on the mental health of women of reproductive age group.

**Materials and Methods:**

A community-based, cross-sectional study was conducted in Raj Nagar- I, urban locality in west Delhi near Palam. 350 women of 15-49 years age group residing in the community were selected by stratified random sampling. These women were administered an interview schedule adapted from WHO multi-country study on women’s health and domestic violence. They were assessed for the presence of domestic violence. Mental health status of these women was estimated by using self-reporting questionnaire 20. Data were analyzed using SPSS 12 software. The test applied was chi square test for proportion and binary logistic regression.

**Results:**

42.8% of the women reported one or the other types of violence. 34.9% of the women reported either physical or sexual violence ever in life. 29.1% of the women reported either physical or sexual violence in past 1 year (current violence). 12% of the women reported mental ill health. Women who had experienced domestic violence were more likely to report mental ill health status and suicidal tendencies as compared to women who had not experienced violence.

**Conclusions:**

Domestic violence is associated with mental ill health.

## Introduction

Violence against women is one of the major public health and human rights problem in the world today. Domestic violence is one of the forms of violence against women. It refers to violence emanating from the household and within relationships covered by familial or emotional (former or present) attachment. It covers physical, sexual and psychological violence occurring in the domestic sphere.(1) It was identified as a major contributor to the global burden of ill health in terms of female morbidity and mortality leading to psychological trauma and depression, injuries, sexually transmitted diseases, suicide and murder.(2) The global health burden from violence against women in reproductive age group is about 9.5 million disability adjusted life years (DALYs).(2) The mental health consequences of domestic violence are alcohol and drug abuse, depression and anxiety, eating and sleeping disorders, feelings of shame and guilt, phobias and panic disorders, physical inactivity, poor self-esteem, post-traumatic stress disorder, psychosomatic disorders, smoking, suicidal behavior and self-harm and unsafe sexual behavior.(3) One of the important obstacles in the prevention of violence against women is the lack of gender sensitive health research and reliable data on the magnitude of the problem and its consequences. This study was conducted with an aim to assess the consequences of violence on the mental health of women.

## Materials and Methods

A community-based, cross-sectional study was conducted in Raj Nagar-I, near Palam, Delhi from April 2005 to March 2006. Raj Nagar has a total population of 9060. The total number of families residing in the area is 2137, the average family size being 4.3. A large majority of the families (95%) are Hindu. Most 250 (71.5%) of the families belong to upper-middle and lower middle socioeconomic strata of society by modified Kuppuswamy scale.(4)

Sampling unit was the household. In each house only one female (selected by simple random sampling, in case more than one) was interviewed. A minimum sample of 10% is usually required. However, since the defined population was less in the present study, so a minimum representative sample of 15% of the households was targeted to be interviewed. Allowing for the possibility of some non-response, 350 households were targeted for interview. The households were chosen by systematic random sampling.

A semi-structured interview schedule adapted from WHO multi-country study on women’s health and domestic violence was used in the study.(5) It was administered on women to check for domestic violence. Domestic violence was assessed as physical, sexual and emotional violence. Physical violence included physically abusive acts like slapping, pushing, hit with fist, kicking, choking and use of weapon. Sexual violence consisted of violent sexual acts like non- consensual sex, physically forced sex and any degrading or humiliating sexual act. Emotional violence was measured by violent emotional acts like humiliation and threatening to hurt. Emotional violence was not analyzed for impact on health. Mental health status of women was assessed by a self-reporting questionnaire (SRQ-20).(5) It involved asking whether the respondent had experienced any of the 20 listed symptoms since past four weeks. The questions were based on symptoms like headache, loss of appetite, feeling of tiredness, problems in digestion, feeling of anxiety, nervousness, loss of interest, difficulty in making decisions, feeling of unhappiness and suicidal thoughts. Eight or more than eight questions answered in affirmative were taken as an indication of unhealthy mental status. Women were interviewed after taking informed consent. The questionnaire was administered by the author (AS) herself.

Data were analyzed using SPSS 12 software. The test applied was chi square test for proportion and binary logistic regression.

## Results

Around 70% of the women in the study population were in the age group of 20-24 and 25-29 years. Around one in three women (31.7%) were married for more than 10 years while 8.3% were married for less than 3 years. Majority of the women (81.7%) were literate and 70.0% of the study population was educated up to middle level and above.

Just about half of the study subjects i.e.150 (42.8%) of the women reported one or the other types of violence. One hundred and twenty two women (34.9%) reported having experienced either physical or sexual violence ever in life. More than a quarter percentage of the women i.e. 102 (29.1%) reported either physical or sexual violence in the past 1 year (current violence).

Forty-two (12.0%) subjects had unhealthy mental status i.e. SRQ ≥ 8. Women with violence were more likely to have unhealthy mental status (21.3%) than without violence (7.0%). The difference was highly significant statistically (*P*=0.0001) [Table 1].

**Table 1:** Assessment of mental health status of women using SRQ-20

Status of mental health	Violence (physical/sexual)		Total
	Present	Absent	
Not healthy	26 (21.3)	16 (7.0)	42 (12.0)
Healthy	96 (78.7)	212 (93.0)	308 (88.0)

Total	122 (34.9)	228 (65.1)	350 (100.0)

*χ*^2^=15.4, df= 1, *P*=0.0001

Mean SRQ score in women who had experienced violence was higher (4.65±4.4) as compared to the women without violence (2.42±2.9). The difference was highly significant (*P*=0.0001).

Logistic regression model was applied and domestic violence was found to be significantly associated with adverse mental health status (OR= 2.9, 95% C.I. 1.4-6.0) after adjusting for age, socio-economic status, duration of marriage and education of the respondent.

22.3% of the study subjects had ever thought of suicide. 12.0% had suicidal thoughts in past 1 month and 3.4% of the women had tried to commit suicide. Suicidal tendencies were more common in women who had experienced violence than those who had never experienced violence and the difference between two groups was statistically highly significant [Table 2].

**Table 2:** Suicidal tendency among women

Suicidal tendency	Violence (physical/sexual)		Total
	Present	Absent	
Suicidal thoughts ever in life			
Yes	45 (36.9)	33 (14.5)	78 (22.3)
No	77 (63.1)	195 (85.5)	272 (77.7)
Suicidal thoughts in past one month			
Yes	25 (20.5)	17 (7.5)	42 (12.0)
No	97 (79.5)	211 (92.5)	308 (88.0)
Tried to commit suicide			
Yes	10 (8.2)	2 (0.9)	12 (3.4)
No	112 (91.8)	226 (99.1)	338 (96.6)

Total	122 (34.9)	228 (65.1)	350 (100.0)

*χ*^2^=23.0, df=1, *P*=0.000; *χ*^2^=12.8,df=1, *P*=0.000; *χ*^2^(Yates corrected) = 10.7, df=1, *P*=0.000

## Discussion

Different studies use different methods for the assessment of domestic violence. For the measurement of domestic violence, WHO has developed a questionnaire which has been used in the WHO multi-country study on women’s health and domestic violence against women in 15 different sites in 10 countries. The same questionnaire was made use of in the present study so that standardization of results could be achieved.

For analyzing the effect of violence on health, effect of ever physical/sexual violence has been considered while emotional violence is often cited as the most hurtful form of violence. There is little agreement on how to capture it in women. For this reason, the association between emotional violence and health status was not analyzed. However, this might mask important health consequences of emotional violence.

Despite the aforementioned limitations, the existing literature depicts an association between violence and mental health. McCauley *et al*. (1995) conducted a study in primary care clinics in Baltimore and found that abused women were significantly more likely to have higher scores on instrument for depression, anxiety, and somatization. They were also more likely to have attempted suicide.(6) Fikree *et al*. (1999) reported statistically significant association between physical abuse and anxiety/depression.(7) Olavarrieta *et al* (2002) found out that currently abused women had higher scores on indicators of depression (*P*<0.001).(8) Kramer *et al*. (2004) reported that abused women were likely to have depression than non abused women (76% *vs* 24%).(9)

A previous Indian study found a significant association between exposure to violence and unhealthy mental status.(10) In another Indian study conducted in five different states, 34.1% of the women suffering from domestic violence reported mental stress, 29.3% reported depression, 26.4% reported disturbed sleep, 21.8% reported anxiety and 15.1% chronic headache.(11)

In WHO multi-country study (2005) also, the mean score for women who had experienced abuse was significantly higher than that for non-abused women. Women who had experienced violence were more likely to have suicidal thoughts (adjusted OR=2.9, 95% C.I 2.7-3.2) and attempted suicide (adjusted OR=3.8, 95% C.I. 3.3-4.5).(12) In our study also, after adjusting for factors like age, socio-economic status, duration of marriage and education of the respondent, those women who were suffering from domestic violence were three times more likely to have adverse mental health outcomes. The findings of the present study match with the observations of the other workers mentioned above and reaffirms that violence is significantly associated with negative mental health outcomes among women.

As the study design is cross-sectional, it will be difficult to assign casualty to domestic violence; hence, further research can be conducted in this direction to assess the temporal nature of association.
